# Ascorbic acid and ascorbate-2-phosphate decrease HIF activity and malignant properties of human melanoma cells

**DOI:** 10.1186/s12885-015-1878-5

**Published:** 2015-11-07

**Authors:** Sarah L. Miles, Adam P. Fischer, Sandeep J. Joshi, Richard M. Niles

**Affiliations:** 1 Department of Biochemistry and Microbiology, Joan C. Edwards School of Medicine, Marshall University, One John Marshall Drive, Huntington, WV 25755 USA; 2 Department of Biochemistry and Molecular Biology, University of Maryland School of Medicine, 655 W. Baltimore Street, Baltimore, MD 21201 USA

**Keywords:** Hypoxia inducible factor-1 alpha, Ascorbic acid, Ascorbate-2-phosphate, Melanoma, Prolyl hydroxylase

## Abstract

**Background:**

Hypoxia inducible factor-1 alpha (HIF-1α) is thought to play a role in melanoma carcinogenesis. Posttranslational regulation of HIF-1α is dependent on Prolyl hydroxylase (PHD 1–3) and Factor Inhibiting HIF (FIH) hydroxylase enzymes, which require ascorbic acid as a co-factor for optimal function. Depleted intra-tumoral ascorbic acid may thus play a role in the loss of HIF-1α regulation in melanoma. These studies assess the ability of ascorbic acid to reduce HIF-1α protein and transcriptional activity in metastatic melanoma and reduce its invasive potential.

**Methods:**

HIF-1α protein was evaluated by western blot, while transcriptional activity was measured by HIF-1 HRE-luciferase reporter gene activity. Melanoma cells were treated with ascorbic acid (AA) and ascorbate 2-phosphate (A2P) to assess their ability to reduce HIF-1α accumulation and activity. siRNA was used to deplete cellular PHD2 in order to evaluate this effect on AA’s ability to lower HIF-1α levels. A2P’s effect on invasive activity was measured by the Matrigel invasion assay. Data was analyzed by One-way ANOVA with Tukey’s multiple comparisons test, or Student-*T* test as appropriate, with p < .05 considered significant.

**Results:**

Supplementation with both AA and A2P antagonized normoxic as well as cobalt chloride- and PHD inhibitor ethyl 3, 4-dihydroxybenzoate induced HIF-1α protein stabilization and transcriptional activity. Knockdown of the PHD2 isoform with siRNA did not impede the ability of AA to reduce normoxic HIF-1α protein. Additionally, reducing HIF-1α levels with A2P resulted in a significant reduction in the ability of the melanoma cells to invade through Matrigel.

**Conclusion:**

These studies suggest a positive role for AA in regulating HIF-1α in melanoma by demonstrating that supplementation with either AA, or its oxidation-resistant analog A2P, effectively reduces HIF-1α protein and transcriptional activity in metastatic melanoma cells. Our data, while supporting the function of AA as a necessary cofactor for PHD and likely FIH activity, also suggests a potential non-PHD/FIH role for AA in HIF-1α regulation by its continued ability to reduce HIF-1α in the presence of PHD inhibition. The use of the oxidation-resistant AA analog, A2P, to reduce the ability of HIF-1α to promote malignant progression in melanoma cells and enhance their response to therapy warrants further investigation.

## Background

Melanoma, a malignancy derived from pigment producing melanocytes found primarily in the epidermis of the skin, continues to be the deadliest form of skin cancer. The global incidence of melanoma is increasing at a faster rate than any other type of cancer. Despite advances in the treatment of metastatic melanoma it remains an incurable disease [1]. The only successful cure for melanoma remains early identification of atypical skin lesions and complete surgical excision before invasion of the deeper dermal tissue [2, 3]. After dissemination and metastasis of the primary tumor, limited treatment strategies have encompassed the use of conventional chemotherapy such as dacarbazine [4, 5], with only slightly more favorable responses with Interleukin-2 (IL-2), interferon–a2b (IFN-a2b) [3, 6] and more recently, mutant BRAF inhibitors and immunostimulants [1, 7, 8]. However, even after initial positive responses, most melanoma tumors become chemoresistant, ultimately leading to treatment failure and refractory disease.

Many factors play a significant role in the initiation and progression of melanoma including genetic alterations, and response to the tumor microenvironment [9–11]. Within the microenvironment, oxygen availability is thought to play a critical role in melanoma carcinogenesis. Hypoxia inducible transcription factor 1 (HIF-1) is a critical mediator of the cellular response to hypoxia. HIF-1 is a heterodimeric complex of α and β subunits. While both the HIF-1α and β subunit mRNAs are constitutively expressed, the HIF-1α subunit protein is tightly regulated through post-translational hydroxylation by oxygen dependent Fe (II)/2-oxoglutarate (2OG) prolyl 4-hydroxylase (PHD) enzymes [12, 13]. This hydroxylation targets HIF-1α ubiquitination and degradation by the proteasome. Under conditions of low oxygen tension, these hydroxylase enzymes are disabled, allowing the stabilization and accumulation of HIF-1α in the cell.

Overexpression and stabilization of HIF-1α has been identified in numerous malignancies [14-17], including melanoma, and has been implicated in driving its progression and metastatic potential [18-23]. Many tumor types including melanoma stabilize HIF-1α under non-hypoxic conditions [24, 18, 20, 25]. HIF acts as a positive regulator of proteins known to be important in melanoma cell invasion, spreading and motility [26]. Given the relationship between HIF-1α and melanoma progression, this transcription factor is an attractive target for small molecule inhibitors [27].

Ascorbic acid (AA) is an essential vitamin in humans due to the evolutionary loss of the gulonolactone oxidase (Gulo) enzyme necessary to catalyze the final step in ascorbic acid synthesis. Its antitumor activity has been studied extensively over the past decades. Numerous *in vitro* and in vivo studies with both human and animal tumors demonstrated correlations between tumor AA levels, reduced HIF-1 activation, and longer disease free survival [28]. Additionally, low AA levels are associated with increased HIF-1 activity and more aggressive tumor phenotypes [29-30]. Furthermore, cancer patients often have depleted reserves of vitamin C [31-34]. AA has varying effects on cancer initiation, progression and growth.

The aim of this study was to assess the effect of physiological concentrations of AA on the normoxic expression and activity of HIF-1α in WM9 metastatic melanoma cells and to determine the mechanism for its action. Because of the potential for off target pro-oxidant effects with the use of high concentrations of ascorbic acid (mM concentrations), our studies aimed to determine whether physiologically attainable serum concentrations of AA [35, 36], which would be achievable through the consumption of vitamin C rich foods (yielding up to 100 μM serum AA) or oral dietary supplements (up to 250 μM serum AA), could effectively impact HIF-1α in melanoma cells [36]. Under normoxic culture conditions, addition of AA to culture media at physiologically relevant concentrations resulted in a rapid reduction of HIF-1α protein, and HIF activity. Interestingly, the transcriptional activity of HIF-1 proved to be more sensitive to AA treatment than the PHD induced degradation of the HIF-1α. We also found that low physiological concentrations of AA were also able to antagonize hypoxia-mimetic (cobalt chloride; CoCl_2_) induced HIF-1α stabilization and increased HIF transcriptional activity. Ascorbate-2-phosphate (A2P), an oxidation resistant analog of AA, was more potent than its parent compound in reducing HIF-1α levels.

## Materials and methods

### Cell culture and reagents

WM1366 and WM9 melanoma cell lines were a generous gift from Dr. Meenhard Herlyn at the Wistar Institute (University of Pennsylvania). Cells were cultured in RPMI 1640 media supplemented with 10 % fetal bovine serum (FBS) and 1 % penicillin/streptomycin, in a humidified 5 % CO_2_ / 95 % air incubator at 37 °C. L-Ascorbic Acid (AA), L-Ascorbic acid 2-phosphate sesquimagnesium salt hydrate (A2P), Cobalt Chloride (CoCl_2_), and Ethyl 3, 4-dihydroxybenzoate (EDHB) were purchased from Sigma Chemical Company.

### Western Blot Analysis and Antibodies

Nuclear protein extracts were isolated using the NePER Nuclear and Cytoplasmic Extraction Kit (Pierce), supplemented with Complete Mini Protease Inhibitor Cocktail (Roche) following the manufacturers protocol. Whole cell lysates were extracted using whole cell lysis buffer (50 mM Tris HCl, 150 mM NaCl, 0.25 % SDS, 0.25 % sodium deoxycholate, 1 mM EDTA; pH 7.4) supplemented with Complete Mini Protease Inhibitor Cocktail (Roche). Equal amounts of protein extracts were separated by SDS-PAGE on 4-20 % MP-TGX precast polyacrylamide gels (BioRad), and transferred to nitrocellulose membrane using the BioRad MINIProtean3 system. Membranes were immunoblotted with antibodies that recognized HIF-1α (1 μl/ml; R&D Systems), EGLN1 (PHD2; 1:1000; Cell Signaling). Anti- β-actin (1:10000; 1 h at RT; Sigma) was used to assess equal protein loading. Immunoblots were visualized using an enhanced chemiluminescence detection kit (ECL Prime; GE Healthcare) and imaged on a PhotoDyne Imaging system (PhotoDyne Technologies). Densitometry was obtained and quantitated using LabQuant Software.

### Small interfering RNA (siRNA) transfection

WM9 cells were transfected at the time of seeding with 10 nM PHD2 siGENOME SMARTpool siRNA (GE Dharmacon; ThermoScientific) or 10 nM non-targeting Control siRNA using Lipofectamine RNAimax (Invitrogen) in standard RPMI culture media following the manufacturers protocol with modifications. Briefly, siRNA transfection complexes were combined in OptiMEM reduced serum media (Invitrogen) following the RNAimax protocol. Cells were removed from the dish via trypsinization, and resuspended in standard RPMI media containing the appropriate siRNA transfection complex. Cells were immediately seeded into 35 mm tissue culture dishes at 1.0x10^5^ cells per dish. Cells were incubated with transfection reagent overnight. Media was replaced the following day with complete RPMI +/− CoCl_2_, EDHB, AA or A2P at the concentrations and times indicated.

### Luciferase reporter assay

2.0X10^5^ cells were seeded in 60 mm culture dishes 24 h prior to transfection. Cells were incubated overnight with transfection mixture containing 1.5 μg HIF-1 pTL-Luc (5’-3’: GTGACTACGTGCTGCCTAGGTGACTACGTGCTGCCTAGGTGACTACGT GCTGCCTAGGTGACTACGTGCTGCCTAG; Affymetrix, LR0128) and 0.1 μg pSV-β-galactosidase plasmids (Clontech) and eXtreme Gene 9 transfection reagent (Roche) following the manufacturers protocol in OptiMEM Reduced Serum media (Invitrogen). Transfection media was replaced with standard RPMI the following day, and cells treated as described. Luciferase activity was measured using the Luciferase Assay Kit (Promega) and normalized against β-galactosidase activity that was measured using the β-galactosidase Assay Kit (Promega). Luciferase and β-gal were measured separately on a SpectraMax M2e 96-well plate reader.

### Matrigel invasion assay

Prior to measuring invasion, WM9 cells were cultured in 10 cm dishes and maintained for 5 days in standard RPMI with or without 100 μM A2P supplementation under standard culture conditions. Invasion was evaluated using BD BioCoat Invasion kits (354481; BD Corning). Cells were dissociated with Accutase (Invitrogen) and 2.5x10^5^ cells were seeded onto the Matrigel coated 6-well inserts and the assay conducted using the manufacturer’s protocol with inclusion of 100 μM A2P supplementation in all media chambers. Plates were incubated 24 h and cells were then fixed with 100 % methanol for 5 min and stained with 0.5 % crystal violet for 5 min before being washed several times in ddH2O to remove excess stain. Inserts were allowed to dry overnight, then membranes were removed from the inserts, and placed in a microtube with 200 μL of 10 % acetic acid for 15 min with vortexing to elute the dye. Sample absorbance was measured in triplicate at 595 nM on a SpectraMax M2e 96 well plate reader. A stained membrane without cells was used as the blank control to account for background staining.

### Reverse transcription (RT) and PCR

Total RNA was isolated from untreated WM9 cells using an RNeasy Mini Kit (Qiagen) following the manufacturers protocol. RNA quality and quantity was assessed spectrophotometrically using a NanoDrop 2000 UV/Vis spectrophotometer. cDNA was synthesized from total RNA using the Advantage RT-for-PCR kit (Clontech Laboratories, Mount View, CA) following the manufacturers recommended protocol. PCR analysis of target sequences were generated using the Advantage cDNA kit (Clontech Laboratories) with the following PCR primers; EGLN1(PHD2): 5’– GGCAAAGCCCAGTTTGCTGAC-3’(forward), 5’ - CCCTCACACCTTTTTCACCTGT-3’ (reverse); EGLN2 (PHD1): 5’- CCAGGCAAGAGAACCAGGAG-3’(forward), 5’-TCAACGTGCCTTACGTACCC-3’ (reverse); EGLN3 (PHD3): 5’– GGCTTCTGCTACCTGGACAACT-3’(forward), 5’- AGGATCCCACCATGTAGCTTG-3’ (reverse). PCR conditions: 95 °C 5 min; 30 cycles of 95 °C 1 min, 57 °C 1 min, 72 °C 1 min; 72° 5 min. PCR products were labeled with Texas Red (Invitrogen), separated on a 1 % agarose gel and visualized by UV using a PhotoDyne Imaging system (PhotoDyne Technologies). Target PCR sequences were normalized to β-actin.

### Statistical analysis

Data were analyzed using GraphPad Prism 6 software (version 6.0f; GraphPad Software, Inc.). Statistical significance was determined by one-way ANOVA followed by Tukey’s multiple comparisons test, or unpaired Student-*t* test as appropriate. Each experiment was performed at least three times and represented as mean ± SEM. p < 0.05 was considered as a significant difference.

## Results

### Ascorbic acid inhibits the normoxic expression of HIF-1α protein in both invasive and metastatic human melanoma cell lines

**Fig. 1:**
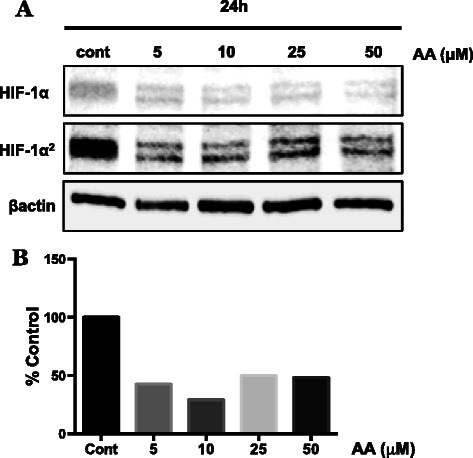
Effect of ascorbic acid on HIF-1α stabilization in WM1366 radial growth phase melanoma cells. WM1366 cells were treated for 24 h with ascorbic acid (AA; 5–50 μM) under standard normoxic culture conditions. **a** Western blot analysis of isolated nuclear extracts reveals considerable reduction in the amount of stabilized HIF-1α protein following treatment with AA. **b** Densitometry analysis demonstrates the ability of AA supplementation at physiologically achievable concentrations to reduce the normoxic overexpression of HIF-1α by approximately 50-60 % in these cells. Protein expression was normalized to β-actin

**Fig. 2:**
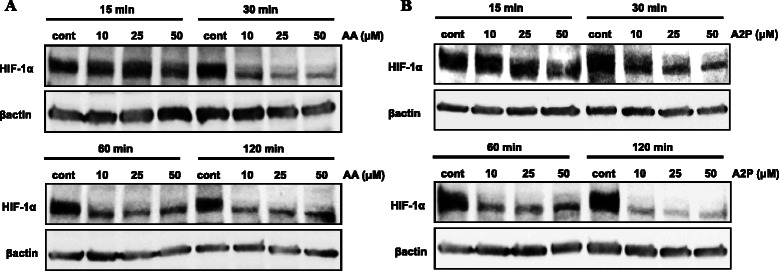
Effect of ascorbic acid and ascorbate 2-phosphate on HIF-1α stabilization in WM9 metastatic melanoma cells. WM9 metastatic melanoma cells were treated with increasing concentrations (10, 25 or 50 μM) of ascorbic acid (AA), or the non-oxidizable analog ascorbate 2-phosphate (A2P) for 15–120 min under standard normoxic culture conditions. Western blot analysis of isolated nuclear fractions reveals that both (**a**) AA and (**b**) A2P cause nearly 50 % reduction of normoxic stabilized HIF-1α in these cells as early as 30 min following treatment. Treatment with A2P provided nearly complete reduction in stabilized HIF-1α by 120 min. Protein expression was normalized to β-actin. All treatments were repeated a minimum of 2 additional times with similar results

We previously reported [26] that when grown under normoxic culture conditions, normal human melanocytes and melanoma cell lines isolated from different stages of the disease had either no detectable HIF-1α protein (normal melanocytes) or increased amounts of HIF-1α protein that roughly correlated with their degree of malignancy. In our initial experiments, we tested the ability of physiological concentrations of ascorbic acid (AA) to decrease the amount of HIF-1α protein in a human melanoma cell line (WM1366), isolated from a vertical growth phase melanoma (Fig. 1a-b). The lowest concentration of AA (5 μM) dramatically decreased the normoxic expression of HIF-1α protein in this human melanoma cell line (Fig. 1B). An increased contrast of the western blot was used to evaluate the signal of the HIF-1α protein by densitometry in the AA treated samples (indicated as HIF-1α^2^ in Fig 1). Using a metastatic human melanoma cell line (WM9), we compared the time and concentration dependent ability of AA or ascorbate-2-phosphate (A2P), an oxidation-resistant analog of AA, to decrease the level of HIF-1α protein (Fig 2). At 15 min, HIF-1α protein is already effectively reduced by approximately 50 % by the 50 μM concentration of AA and A2P. However, within 30 min, the amount of HIF-1α protein was decreased by >50 % by all concentration in both the AA and A2P-treated WM9 cells. The decrease was maintained during the 2 h time period. A2P treatment most effectively reduced HIF-1α protein levels below 15 % by 2 h.

### Ascorbic acid and A2P also inhibit hypoxia-mimetic-induced HIF-1α protein stabilization

**Fig. 3:**
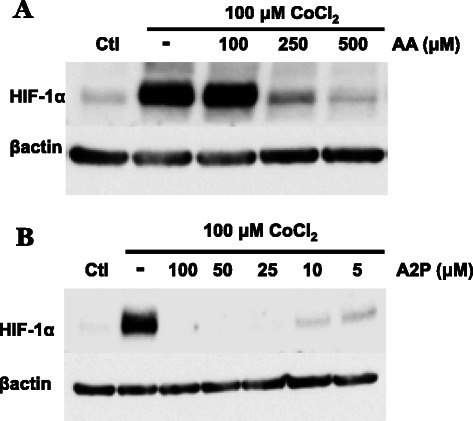
Effect of AA and A2P on cobalt chloride induced HIF-1α protein accumulation in metastatic melanoma. WM9 metastatic melanoma cells were treated for 24 h with the hypoxia mimetic cobalt chloride (100 μM) in the presence or absence of AA and A2P and nuclear extracts analyzed by western blot. **a** The addition of AA (100–500 μM) reveals that 100 μM AA is unable to reduce CoCl_2_ induced HIF-1α accumulation. Higher concentrations of AA (250 and 500 μM) are necessary to reduce induced levels of HIF-1α **b**, while cells treated with A2P (5.0-100 μM) show that A2P efficiently reduces CoCl_2_ induced accumulation of HIF-1α at concentrations as low as 5 μM. Protein expression was normalized to β-actin. All treatments were repeated a minimum of 2 additional times with similar results

It was important to determine whether AA and A2P could also decrease the much higher levels of HIF-1α found in hypoxic regions of tumors. We used CoCl_2_ as a mimetic of hypoxia since this induced a minimum 15–20 fold increase in WM9 HIF-1α protein level (Fig. 3). Treatment of CoCl_2_-treated cells with different concentrations of AA or A2P revealed that the latter compound was also substantially more effective in reducing the amount induced HIF-1α protein. Since 100 μM A2P eliminated essentially all of the HIF-1α protein, compared to 100 μM AA, which at this concentration had no effect on the protein, we titrated down the amount of A2P and found that 5 μM still reduced CoCl_2_ induced HIF-1α by 90 % (Fig. 3).

### Ascorbic acid and A2P inhibit HIF transcriptional activity in metastatic human melanoma

**Fig. 4:**
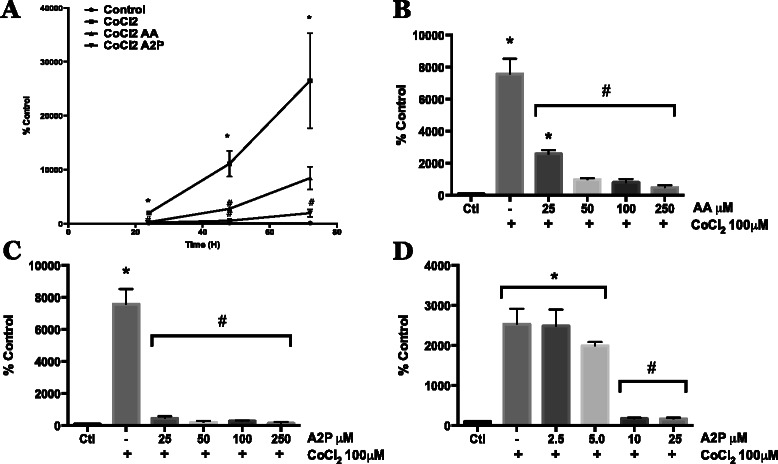
Effect of AA and A2P on HIF-1α transcriptional activity in metastatic melanoma. WM9 metastatic melanoma cells were transiently transfected with an HIF-1 HRE-luciferase reporter vector. **a** Transfected WM9 cells were treated with 100 μM CoCl2 with or without AA (100 μM) or A2P (100 μM) Cells were collected and HIF-1 transcriptional activity was measured by luciferase assay at 24, 48, and 72 h. Both AA and A2P significantly reduced HIF-1 transcriptional activity at 24 and 48 h, at 72 h, A2P significantly reduced CoCl_2_ induced activity while AA began to show reduced efficacy by 72 h. **b** Dose dependent inhibition of CoCl_2_ induced HIF-1 reporter activity using 25, 50, 100 and 250 μM AA. AA significantly reduced CoCl_2_ induced HIF-1 activity at all concentrations, with 25 μM AA beginning to show reduced efficacy. **c** Dose dependent inhibition of CoCl_2_ induced HIF-1 reporter activity by 25, 50, 100 and 250 μM A2P. All concentrations of A2P significantly reduced CoCl_2_ induced HIF-1 reporter gene activity. For this reason, lower doses of A2P were then tested. **d** Low dose titration of A2P dependent inhibition of CoCl_2_ induced HIF-1 reporter activity using 2.5, 5.0, 10 and 25 μM A2P. A2P demonstrated close to maximum inhibition of HIF-1 activity at concentrations as low as 10 μM, with 5 and 2.5 μM demonstrating little to no inhibition of activity. All HRE-luciferase activity was normalized to β-galactosidase activity. Data are represented as mean ± SEM of a minimum of n = 3, analyzed by One-way ANOVA followed by Tukey’s multiple comparisons test; * denotes significant difference from control, p < 0.0001, # denotes significant difference from CoCl_2_ treatment alone, p < 0.003-0.0001

While high levels if HIF-1α imply induction of hypoxia-inducible genes, this needs to be verified by measuring HIF transcriptional activity. Therefore, we used a HIF-luciferase reporter plasmid transfected into WM9 metastatic human melanoma cells to examine the influence of AA and A2P on this activity. The hypoxia-mimetic CoCl_2_ induced a time dependent increase in HIF activity and this was antagonized by both 100 μM AA and A2P, with the latter compound being more effective (Fig. 4a). We also measured the concentration-dependent ability of AA and A2P to inhibit CoCl_2_-induced HIF activity (Fig. 4 b,c,d). AA at 25 μM inhibited HIF activity by ~70 % with 50–250 μM causing a further decrease in activity (Fig. 4b). In contrast, 25 μM A2P eliminated >90 % of the HIF activity (Fig. 4c). Due to this potent effect, we treated the WM9 cells with 2.5-20 μM A2P. There is a significant decrease (>95 %) in HIF activity at a concentration of 10 μM A2P (Fig. 4d).

### PHD and AA/A2P inhibition of HIF activity

**Fig. 5:**
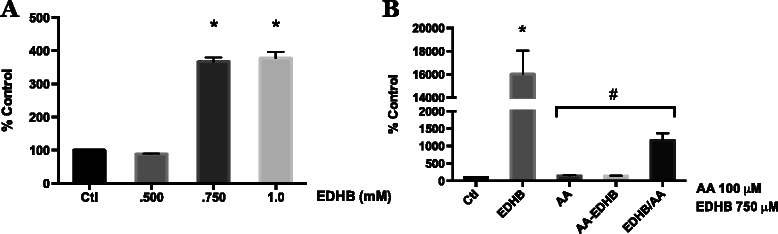
Effect of ascorbic acid on EDHB induced HIF-1 transcriptional activity in melanoma cells. WM9 metastatic melanoma cells were transiently transfected with an HIF-1 HRE-luciferase reporter vector. **a** Transfected cells were treated for 24 h with 0.5, 0.75 and 1.0 mM EDHB. Induction of HIF-1 transcriptional activity was measured by luciferase assay. EDHB at 0.75 mM was found to be the lowest dose capable of generating near-maximal induction of HIF-1 transcriptional activity and was thus chosen for subsequent experiments. **b** Cells were treated for 24 h with 0.75 mM EDHB alone (EDHB), 100 μM AA alone (AA), pretreated with 100 μM AA for 4 h prior to addition of EDHB (AA-EDHB), or treated with100 μM AA and EDHB concurrently (EDHB/AA). AA effectively reduces EDHB induced HIF-1 transcriptional activity; with AA pretreatment showing increased efficacy at inhibiting EDHB induced HIF-1 activity vs. concomitant treatment. All HRE-luciferase activity was normalized to β-galactosidase activity. Data are represented as mean ± SEM of n = 3, analyzed by One-way ANOVA followed by Tukey’s multiple comparisons test; * denotes significant difference from control, p < 0.0001, # denotes significant difference from EDHB treatment alone, p < 0.0001

**Fig. 6:**
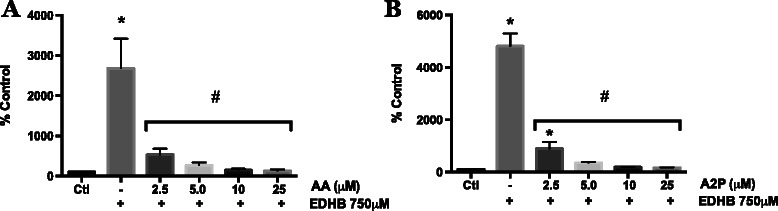
Effect of AA and A2P on EDHB induced HIF-1 transcriptional activity in melanoma cells. WM9 metastatic melanoma cells were transiently transfected with an HIF-1 HRE-luciferase reporter vector. Transfected cells were treated for 24 h with 750 μM EDHB in the presence of **a** 2.5, 5.0, 10 or 25 μM AA or **b** 2.5, 5.0, 10 or 25 μM A2P. HIF-1 transcriptional activity was measured by luciferase assay. Data are presented as the mean ± SEM of n = 3, analyzed by One-way ANOVA followed by Tukey’s multiple comparisons test; * denotes significant difference from control, p < 0.0001, # denotes significant difference from EDHB treatment alone, p < 0.0001

AA serves as an essential co-factor for PHD enzyme activity that results in the targeted degradation of HIF-1α. Also AA levels are frequently lower in human tumors than in the surrounding uninvolved tissue [28]. Therefore we investigated whether AA and A2P decreased HIF-1α protein in WM9 metastatic melanoma cells through acting on PHD. First we examined whether AA and A2P could counteract the effect of the PHD inhibitor ethyl 3,4-dihydroxybenzoate (EDHB). Figure 5 illustrates the concentration dependent ability of EDHB to stimulate HIF reporter gene activity in WM9 cells. The maximum stimulation was achieved at 750 μM and this concentration was used in all further experiments. EDHB is thought to inhibit PDH activity through both binding to the AA site on the enzyme and by chelation of iron. Since AA has iron chelating activity, we tested the ability of AA to reverse the EDHB stimulation of HIF activity when added prior to EDHB treatment or when the two compounds were simultaneously added to the cells. Clearly, AA is more effective in reversing EDHB stimulation of HIF activity (through inhibition of PHD activity) when pre-incubated with the cells (Fig. 5 right panel, AA-EDHB) vs. adding it at the same time as EDHB (Fig. 5 right panel, EDHB/AA). Next, we compared the concentration-dependent ability of A2P vs. AA to reverse the EDHB-stimulated HIF activity (Fig. 6). In contrast to the superior ability of A2P vs. AA to inhibit cobalt chloride induced HIF-1α levels (Fig. 4), the potency of A2P and AA to inhibit the EDHB-induction of HIF transcriptional activity was similar.

**Fig. 7:**
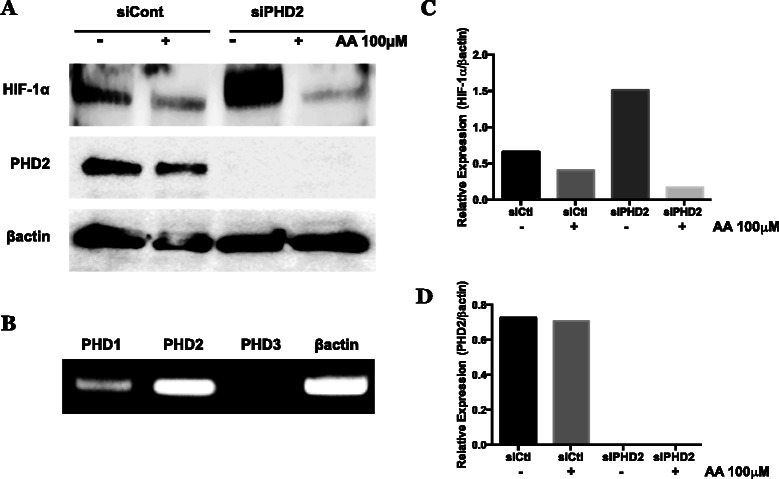
Effect of PHD2 knockdown on reduction of normoxic HIF-1α protein by AA in metastatic melanoma. WM9 metastatic melanoma cells were transfected using non-targeting control siRNA or siGENOME SMARTpool siRNA against PHD2. **a** siRNA transfected cells were treated for 24 h with or without 100 μM AA under standard normoxic culture conditions. HIF-1α and PHD2 were analyzed by western blot, and normalized to β-actin. Knockdown of PHD2 caused an increase in stabilized HIF-1α protein, however, does not result in loss of effectiveness of AA to reduce accumulated HIF-1α. **b** qPCR analysis of PHD1, 2, and 3 isoforms in untreated WM9 metastatic melanoma cells, normalized to β-actin expression. PHD2 appears is the prevalent isoform, however the presences of PHD1 may contribute to the retained activity of AA following PHD2 selective knockdown. **c, d** Densitometry analysis of HIF-1α and PHD2 expression following PHD2 knockdown and AA treatment. siRNA experiments were repeated a minimum of 2 additional times with similar results

To more directly determine whether PHD was the target that mediated the ability of AA to decrease HIF-1α protein levels, we first determined which PHD isoforms were expressed in the WM9 cells. RT-PCR analysis shows that PHD2 had the highest RNA expression followed by PHD1 (Fig. 7b). We could not find any RNA expression of PHD3. We used PHD2 siRNA to knock-down the expression of PHD2. The amount of PHD2 protein was reduced by greater the 90 % in cells treated with the siPHD2 relative to cells treated with a control siRNA (Fig. 7a, d). Despite this dramatic decrease in PHD2 protein, AA treatment of the cells still markedly decreased HIF-1α protein levels. Note that the amount of HIF-1α is much higher in the siPHD2 vs. control siRNA treated cells likely due to the absence of PHD2 (Fig. 7a, c).

### A2P inhibits human metastatic melanoma cell invasion *in vitro*

**Fig. 8:**
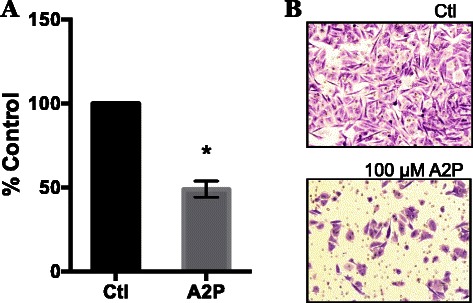
Effect of A2P treatment on invasive potential of metastatic melanoma cells. WM9 metastatic melanoma cells were maintained in 100 μM A2P for 5 days under standard normoxic culture conditions. Cells were seeded into Matrigel chambers and assayed for invasion after 24 h. **a** Matrigel invasion assay was completed as described in Methods and Materials. Cells grown in the presence of A2P demonstrated a 50 % reduction in invasion. **b** Representative photographs of Matrigel invasion chambers. Data are represented as mean ± SEM of *n* = 3, analyzed by Student paired *T*-test; * denotes significant difference from control, p < 0.0087

Clinical samples of human melanoma express high levels of HIF-1α [19, 37]. Also, siRNA knockdown of HIF-1α decreases invasion through Matrigel [25]. Therefore we tested whether A2P would also decrease the invasion of WM9 human metastatic melanoma cells. Using the Matrigel *in vitro* invasion assay (Fig. 8) we found that treatment of the WM9 cells with A2P decreased invasion by 50 %.

## Discussion

Once melanoma progresses to the invasive and metastatic stage, it is very difficult to treat. Therefore, it is important to identify the molecular changes that contribute to the malignant progression of this disease. Hypoxia and acquisition of a vascular network together with reprograming of the cancer cell’s metabolism have been noted as important events required for tumor progression [38, 17]. Accumulation of HIF-1α and HIF-2α was measured via immunohistochemistry in 46 patient samples of nodular cutaneous malignant melanomas [19]. Expression of HIF-1α and HIF-2α was directly correlated with vascular endothelial growth factor accumulation (VEGF) and also associated with poor prognosis. A later study of 89 patients with primary cutaneous melanoma did not show a correlation between

HIF-1α and overall survival or disease-free survival [20]. However, the relative amount of HIF-1α and more importantly the activity of HIF as assessed by target gene expression in the samples was not assayed. HIF-1α was found under normoxic conditions in malignant melanoma cells, but not in normal human melanocytes. Further, the amount of HIF-1α was increased in cells from invasive and metastatic human melanomas relative to that found in cells from radial growth phase melanomas. Also knockdown of HIF-1α in the metastatic cells led to marked decrease in anchorage-independent growth and the ability to invade through Matrigel [25]. Kuphal et al. [18] verified the constitutive expression of HIF-1α in malignant melanoma and their studies implicate ROS and the NF-κB pathways in contributing to this accumulation of HIF-1α.

Due to their role in tumor progression, HIF-1α and HIF are targets for the development of new small molecular inhibitors. However, most of the inhibitors to date work in an indirect fashion such as Bortezomib, a proteasome inhibitor and geldanamycin a Hsp90 inhibitor [39] In fact, medicinal chemists have deemed that HIF is undrugable [39].

AA (vitamin C) plays a direct role in regulating both the activity of PHD and thus the stability of HIF-1α and the activity of FIH, which inhibits the transcriptional activity of HIF. There are several reports that addition of AA to cancer cell lines decreases the amount of HIF-1α protein and also inhibits HIF activity [40–42]. Thus AA might be useful as a direct inhibitor of the HIF pathway presumably through its action on the family of Fe (II)-2-oxoglutarate-dependent oxygenases, of which PHD and FIH are members. We investigated this possibility in human malignant melanoma cells.

In agreement with the study of Knowles, et al. [40] we found that AA decreased the amount of HIF-1α protein in malignant melanoma cells grown under either normoxic or hypoxic-mimetic (CoCl_2_) conditions (Figs. 1, 2 and 3). Further, we showed that A2P, a less oxidizable analog of AA, was more potent than AA in reducing the amount of HIF-1α in the melanoma cells treated with CoCl_2_ (Figs. 2 and 3). We could not find any other reports on the effect of A2P on HIF-1α levels, but several studies show that A2P inhibits tumor invasion [43, 44], while it also inhibits melanogenesis in melanocytes [45]. A2P was also more potent than AA in reducing HIF reporter gene activity (Fig. 4 panels B & C). Also note that the ability of AA to inhibit HIF reporter gene activity was more potent than its ability to decrease HIF-1α protein levels (compare Fig. 4B with Fig. 3). This finding agrees with the report of Kuiper et al. [41] that AA preferentially suppresses the HIF-1 transcriptional response. The authors suggest that this preference is likely due to AA’s ability to stabilize and reduce the iron atom in the PHD and FIH active sites, with FIH (asparagine hydroxylase) being more sensitive to fluctuations in intracellular ascorbate.

Next we investigated the hypothesis that the effect of AA on decreasing HIF-1α in human melanoma cells was mediated through stimulation of PHD and or FIH activity. First we used a pharmacological inhibitor of PHD and likely FIH, ethyl-3, 4-dihydroxybenzoate (EDHB). This inhibitor decreases prolyl hydroxylase activity [46] through both competition for the AA binding site [47] and inducing an iron deficiency state in cells through a low affinity for ferric iron [48]. In our melanoma cells, EDBH at or above a concentration of 750 μM stimulated HIF reporter gene activity by 4-fold. Since AA pretreatment was more effective in blocking the EDHB stimulation of HIF-reporter gene activity than when EDHB and AA were add simultaneously to the cells, we suggest that there is competition for either the ferric iron or the AA binding site on the PHD/FIH enzymes (Fig. 5).

After defining the condition for maximum inhibition of PHD by EDBH as measured by HIF-reported gene activity, we then measured the concentration-dependent ability of pre treatment with either AA or A2P to reverse the inhibition as determined by a decrease in HIF-reporter gene activity (Fig 6). In contrast to the hypoxia mimetic CoCl_2_ stimulation of HIF-reporter gene activity where A2P was more potent than AA in reversing this stimulation, AA and A2P were similar in their potency for reversing EDHB stimulation of HIF-reporter gene activity. This may be due to off-target (non-PHD) effects of CoCl_2_ causing greater stimulation of HIF-reporter gene activity relative to the activity induced by the PHD selective inhibitor, EDHB which may be more sensitive to AA supplementation.

Since chemical inhibitors can have off-target effects, we used siRNA to knock down the expression of the PHD2 isoform protein. An RT-PCR survey of the expression of PHD isozymes in the WM9 cells revealed that these cells express predominantly PHD2 and a small amount of PHD1, but we could not detect expression of PHD3. The PHD2 isozyme contributes the majority of the HIF-hydroxylase activity in cells with normal oxygen levels [49, 50]. Since PHD1 is localized exclusively in the nucleus [51], it should only be able hydroxylate HIF-1α after it has been stabilized and transported into the nucleus. We were able to knockdown the expression of PHD2 in normoxic WM9 cells by greater than 90 %. As shown in Fig. 7, this knockdown resulted in a 2.3 fold higher amount of HIF-1α relative to cells treated with the control siRNA. Despite the knockdown of PHD2 and the increase in the level of HIF-1α, the addition of 100 μM AA still decreased the amount of HIF-1α by nearly 90 %. There are at least two explanations for this unexpected result. One is that AA has additional modes of action, other than affecting prolyl hydroxylase, which result in a decrease in the HIF-1α protein. The other possibility is that in the absence of PHD2, the isozyme PHD1 can be stimulated by AA and result in the targeting of HIF-1α for degradation by the proteasome.

Regardless of the mechanism for the ability of AA and A2P to decrease HIF-1α levels and inhibit HIF transcriptional activity, the important question is whether blocking the HIF pathway decreases some of the malignant properties of the WM9 metastatic melanoma cells. We addressed this question by measuring the ability of WM9 cells to invade through Matrigel. A2P was able to inhibit invasion by 50 %. A2P did not inhibit the proliferation of WM9 cells (data not shown). These findings, together with our previous work [26] demonstrating that siRNA knockdown of HIF-1α also inhibits invasion through Matrigel, suggests that AA affects the invasive ability of these metastatic cells through a decrease in HIF-1α/HIF activity.

Although our studies used established human melanoma cell lines, there are some in vivo studies that link AA levels to tumor aggressiveness. Low AA levels are associated with increased HIF-1α levels and HIF stimulated gene products in human endometrial tumors [29]. In contrast, increased tumor AA is associated with longer disease-free survival and decreased HIF-1α and HIF stimulated gene products in human colorectal tumors. Specifically in melanoma there is decreased plasma ascorbate levels in stage IV melanoma patients [31] while an epidemiological study found an association between dietary vitamin C (AA) and the risk of cutaneous melanoma in a Northern Italian population [52]. IL-2 treatment of melanoma is unfortunately associated with severe toxicity and it causes a large decrease in circulating levels of AA. A clinical trial has been proposed to assess the use of intravenous AA as an adjuvant to IL-2 treatment of melanoma [53]. Thus AA has many potential roles and uses in human melanoma. The next step will be pre-clinical investigations of AA/A2P and HIF-1α/HIF activity in animal models that most closely recapitulate the initiation and progression of human melanoma.

## Conclusion

Our studies suggest a positive role for ascorbic acid in regulating HIF-1α in melanoma. The addition of ascorbic acid can effectively reduce the amount of stabilized HIF-1α found under normoxic conditions in both vertical growth phase WM1366 and WM9 metastatic melanoma cells. The addition of ascorbic acid also significantly reduces the transcriptional activity of HIF-1α in WM9 metastatic melanoma cells, resulting in decreased invasive potential. Our data supports the function of AA as a critical cofactor for PHD, restoring PHD function to reduce protein accumulation, and likely FIH activity resulting in significant reduction of HIF-1α transcriptional activity. However, there may also be non-PHD mediated mechanisms by which AA reduces the level of the HIF-1α protein. The overexpression of intra-tumor HIF-1α, as well as ascorbic acid deficiency has been noted not only in melanoma, but in other tumor types as well. Further studies to evaluate the causes of ascorbic acid deficiency and its role in the loss of HIF-1α regulation in malignancy are needed. The use of ascorbic acid as a non-toxic adjuvant therapy to aid in the inhibition of HIF-1α activity in order to reduce tumor progression and improve patient response to clinical therapy warrants further investigation.

## Abbreviations

2-OG: 2-oxoglutarate
AA: Ascorbic acid
A2P: Ascorbate 2-phosphate
CoCl_2_: Cobalt chloride
EDHB: Ethyl 3, 4-dihydroxybenzoate
FIH: Factor Inhibiting HIF
HIF-1α: Hypoxia Inducible Factor 1-alpha
HRE: Hypoxia response element
PHD: Prolyl hydroxylase
